# Do We Need to Delineate the Humeral Head in Breast Cancer Patients?

**DOI:** 10.3390/cancers14030496

**Published:** 2022-01-19

**Authors:** Lahcene Belaidi, Pierre Loap, Youlia Kirova

**Affiliations:** Department of Radiation Oncology, Institut Curie, 26 rue d’Ulm, 75005 Paris, France; pierre.loap@gmail.com (P.L.); youlia.kirova@curie.fr (Y.K.)

**Keywords:** breast, radiotherapy, humeral head, helical tomotherapy, late adverse events

## Abstract

**Simple Summary:**

Humeral heads can be unintentionally exposed during breast radiotherapy, particularly when regional lymph nodes are targeted. The aim of this study was to analyze late adverse events involving the humeral head occurring after adjuvant locoregional breast radiotherapy. We included 159 breast cancer patients locoregionally irradiated in an adjuvant setting with helical tomotherapy. After a median delay of 48 months, 10 patients (6.06% (3.20–10.92%) of breasts treated) presented clinical events such as localized bone pain, shoulder functional limitation and humeral head fracture. The average mean and maximum doses to humeral heads were 9.18 Gy and 24.41 Gy, respectively, and were not statistically associated with humeral head adverse events. We found that clinical adverse events involving the humeral head after adjuvant helical tomotherapy for breast cancer were rare, and the radiation exposure was low. No correlation was found between dosimetric parameters and late toxicity.

**Abstract:**

Humeral heads can be unintentionally exposed during breast radiotherapy, particularly when regional lymph nodes are targeted. Moreover, rotational intensity-modulated radiation therapy techniques, such as helical tomotherapy (HT), increase the low-dose bath, the consequences of which are subject to debate. The aim of this study was to analyze late adverse events involving humeral heads occurring after adjuvant locoregional breast radiotherapy with HT. This single-center retrospective study included 159 breast cancer patients locoregionally irradiated, including the regional lymph nodes, in an adjuvant setting with HT at Institut Curie (Paris, France), between January 2010 and 2016. After a median delay of 48 months, six patients (3.8%) developed localized bone pain, three (1.9%) developed a shoulder functional limitation and one (0.6%) developed a traumatic humeral head fracture. The average mean and maximum doses to humeral heads were 9.18 Gy and 24.41 Gy, respectively, and were not statistically associated with humeral head adverse events. Adverse events were statistically more frequent after mastectomy than after breast-conserving surgery. Berg’s level 1 and 2/3 irradiation, and right-sided radiotherapy were associated with an increased maximum dose. In summary, clinical adverse events were rare, and radiation exposure to humeral heads was low. No correlation was found between dosimetric parameters and late toxicity.

## 1. Introduction

Doses received by humeral heads during locoregional breast radiotherapy, including the lymph node areas, as well as their clinical impact, constitute a subject that has been little studied in the literature. However, ESTRO recommendations in force, published in 2015 [1], recommend a delineation of these humeral heads and a minimum distance of 1 cm between humeral heads and the Berg L1 lymph node station’s clinical target volume (CTV).

Moreover, in recent years, we have seen a rapid increase in indications for treatment via conventional intensity-modulated radiotherapy (IMRT) in breast cancers [2,3,4]. These techniques present many advantages and allow better target volume coverage, with a limitation on doses received by organs at risk. However, to accomplish this, they use a multitude of beam entry angles, inevitably accompanied by an increase in the low-dose bath.

The consequences of this increase in the overall dose are now a topic of debate.

Abbassi et al. [5] showed that the average dose received by the lower axillary lymph node (Berg’s level 1) when it was not intentionally treated was non-negligible, and greater with treatment via helical tomotherapy (44.4 Gy) and VMAT (41.0 Gy) than via classic 3D-CRT (30.8 Gy).

Within this context, we wanted to carry out a study on the clinical consequences in terms of the humeral heads of a breast and lymph node station irradiation via helical tomotherapy.

The primary aim was to describe the doses received as well as the clinical events likely to result from this radiotherapy.

The secondary aim was to search for predictive factors of the doses received and clinical events.

## 2. Materials and Methods

### 2.1. Population

This retrospective study was conducted in Institut Curie’s radiotherapy oncology department in Paris. It included all patients treated via adjuvant helical tomotherapy, after breast-conserving surgery (BCS) or total mastectomy, between January 2010 and January 2016. Patients suffering from metastatic disease, those with breast implants and those who had not been treated in the lymph nodes were excluded from the analysis. Population characteristics were published in a prior study [3].

The study was approved by the institutional review board of Institut Curie (The Breast Cancer Research and Treatment Board of Institut Curie).

### 2.2. Terms of the Treatment

The prescribed dose was 50 Gy in 25 fractions over the entire breast or the chest wall, and on the lymph nodes (level L4 + internal mammary nodes (IMN) +/− L3, L2 and L1). A boost from 10 to 16 Gy on the tumor base could also be applied in accordance with institutional recommendations.

All the planning scans were taken from the tragus to the intervertebral foramen L2/L3, without the injection of a contrast agent, using a Toshiba Aquilion LB scanner (Toshiba Corp., Minato, Japan). The width of the sections was 3 mm.

The patients were placed on their back using the AIO positioning system (ORFIT, Wijnegem, Belgium) on a surface at a 5° angle. Their arms were placed above the head, and an immobilization system was placed under the knees.

The scanner images were transferred to a contouring software (Eclipse 3D version 13.6; Varian Medical Systems Inc., Palo Alto, CA, USA). Target volume delineation was performed in accordance with the ESTRO recommendations in force [1].

The anatomic humeral heads were delineated on simulation scanners in the treatment position.

The contoured images were then transferred to the tomotherapy machine’s planning system (TomoTherapy Hi-ART version 3.1.2.3; Tomotherapy Inc., Madison, WI, USA).

### 2.3. Data Collection

Dosimetric data were collected via analysis of dose–volume histograms, and those for clinical events suspected to be connected to the humeral head (shoulder pain, impaired mobility and proximal humerus fracture) were obtained by reading computerized records. During the first 5 years following treatment, patients who had received chemotherapy were examined in consultation every 4 months, and those who had not received chemotherapy every 6 months. After the first 5 years, the follow-up was annual.

### 2.4. Statistical Analysis

Statistical analyses were conducted using the R software version 4.0.1.

Maximum and mean dose averages were estimated with a confidence interval of 95%.

The comparative tests of the doses received were performed using a Mann–Whitney U test for categorical variables and a Spearman correlation test for quantitative variables. Multivariate analysis was carried out using a multiple linear regression.

Comparative tests of the clinical events’ frequency were performed using Fisher’s exact test for categorical variables and logistic regression for quantitative variables.

The statistical significance threshold was set at 0.05.

## 3. Results

### 3.1. Population Description

**Figure 1:**
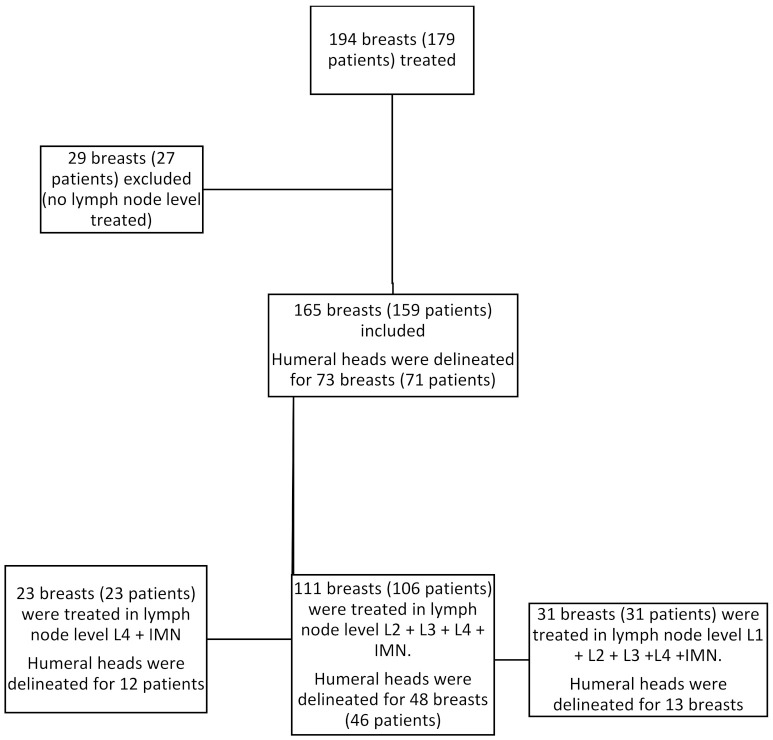
Flowchart describing the number of patients and breasts treated according to lymph node station.

A total of 179 patients (194 breasts) were treated via adjuvant tomotherapy to the breast or chest wall. Among these, 159 patients (165 breasts) received treatment in the lymph nodes. A total of 23 patients (23 breasts) were irradiated at the Berg level 1 axillary lymph node and the internal mammary lymph node chain (IMLN). A total of 106 patients (111 breasts) were treated in the L2, L3 and L4 lymph nodes and IMLN. Finally, a total of 31 patients (31 breasts) were treated in the L1, L2, L3 and L4 lymph nodes and IMLN (Figure 1).

The humeral heads were contoured for 71 patients (73 breasts). For the needs of this study, some humeral heads were delineated retrospectively

The median age was 53 (range: 25–76). In this population, 134 patients (81%) had been treated via conservative surgery, and 31 (19%) had received a mastectomy.

There were 129 patients (78%) who had received axillary lymph node dissection (ALND). A total of 52% of the irradiations were on the right side. The median BMI was 25.7 kg/m^2^ (range: 16.71–53.41).

Additional information on population characteristics was published in a prior study [3].

### 3.2. Humeral Head Radiation Exposure

**Figure 2:**
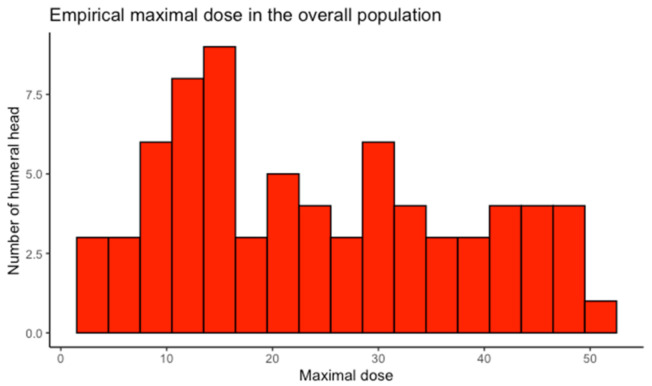

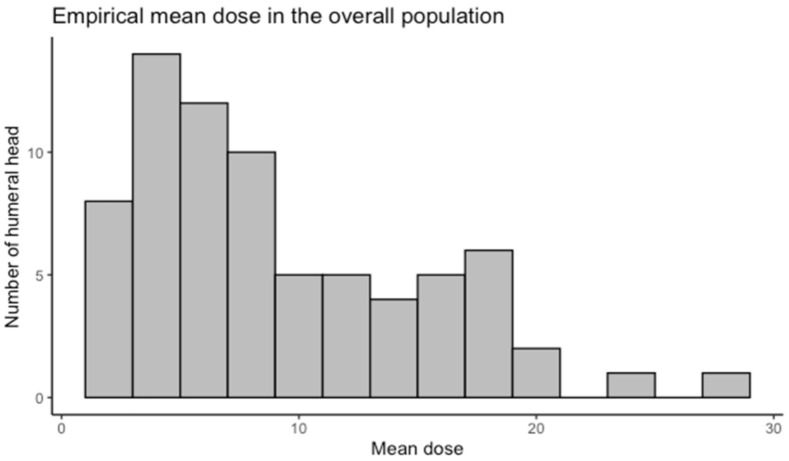
Empirical distribution of maximum and average doses received by the humeral head.

The maximum and average doses received for the 73 patients whose humeral heads had been contoured were, on average, 24.41 Gy (21.20–27.61) and 9.18 Gy (7.91–10.54) (Table 1), respectively. A histogram to assess the distribution of these doses is presented in Figure 2. The maximum doses ranged from 3.53 to 52.20 Gy, and the average doses from 1.83 to 27.57 Gy.

Irradiation of lymph nodes L2 + L3 + L4 + IMLN was associated with a maximum dose that was significantly higher than that of irradiation of lymph nodes L4 + IMLN alone: 24.72 Gy (21.09–28.35) vs. 10.99 Gy (7.49–14.49) (*p* < 0.001). Irradiation of lymph nodes L1 + L2 + L3 + L4 + IMLN was associated with a maximum dose that was significantly higher (35.64 Gy (27.62–43.66)) than that of irradiation of lymph nodes L2 + L3 + L4 + IMLN (*p* < 0.01).

The maximum dose was also, on average, higher with treatment of the right side than with treatment of the left side: 28.24 Gy (23.47–33.01) vs. 21.25 Gy (16.98–25.51) (*p* = 0.034), respectively.

In multivariate analysis (including irradiation of L2/L3 lymph nodes and L1 lymph nodes, and side treated), these three factors continued to be significantly associated with the maximum dose.

### 3.3. Clinical Outcomes

With a median follow-up of 5.6 years (1.2–117 months) from the start of radiotherapy, 10 patients (6.06% (3.20–10.92%) of breasts treated) presented a clinical event in connection with the humeral head, defined as shoulder pain (6 patients, 3.8%), impaired mobility (3 patients, 1.9%) or proximal humerus fracture (1 patient, 0.6%).

The median timeline for the occurrence of clinical events was 48 months (range: 6–99) (Table 2).

In univariate analysis, the only factor significantly associated with clinical event occurrence was the type of surgery carried out (*p* = 0.032), with a higher proportion of symptomatic patients in cases of mastectomy (16.12% (5.4–33.72%)) than in cases of conservative surgery (3.73% (1.22–8.49%)). There was no link with lymph node areas treated, with ALND, with age or with BMI (Table 3). Patients presenting with a clinical event had not, on average, received a maximum or average dose greater than that of those not presenting with any clinical events (*p* = 0.74 and 0.39, respectively). A second analysis including only pain and proximal humerus fracture as clinical events also did not show any significant difference in the maximum and average doses between symptomatic and asymptomatic patients.

## 4. Discussion

Clinical events in connection with the shoulder are relatively rare in the 6 years following breast and lymph node radiotherapy via tomotherapy, and these events do not appear to be associated with the dose received in the humeral head. This study is one of the first asking this question.

There seems to be a trend towards an increased frequency of these events in irradiation of lymph node areas L2/L3 and L1, but it is not statistically significant.

The only significantly correlated factor is mastectomy, suggesting an origin linked to surgery rather than radiotherapy. Thus, the data from this study are globally reassuring concerning breast radiotherapy toxicity in connection with the dose to the humeral heads.

Because many institutions still commonly use three-dimensional conformal radiotherapy techniques in breast cancer, the number of humeral head-related events is relatively low in the clinic. These colleagues are probably still not interested in the asked question, but with the increased use of IMRT, it could rapidly become a pertinent one.

Indeed, in recent years, numerous publications have shown the benefits of IMRT in breast cancer in terms of reduced toxicity [6,7,8,9,10,11,12,13,14]. These techniques are increasingly used for breast cancer treatment, particularly in patients with a difficult anatomy and/or complex treatment volumes [15,16,17,18,19]. As they carry a greater risk of a dose being applied to the humeral head [2,3,4,5], it is necessary to address whether or not this has clinical significance, and if so, to provide guidelines to radiation oncologists for treatment planning and optimization for humeral head tolerance doses. If not, no delineation is needed.

In the literature, data related to dose restrictions for these humeral heads are somewhat limited. In 2007, Dogan et al. [20] took, as a restriction, a maximum dose of 40 Gy, but this choice was made arbitrarily. In our patient cohort, this restriction was exceeded in 20% of the breasts treated (54% of treatment plans included L1 + L2 + L3 + L4 + IMLN, 16% of treatment plans included L2 + L3 + L4 + IMLN, 0% of treatment plans included L4 + IMLN). Only 2 symptomatic patients out of the 10 (20%) received a maximum dose higher than 40 Gy.

Concerning the specific risk of fracture after irradiation of the bony tissue, the data are also piecemeal.

Although the context is not the same and therefore the extrapolation is up for debate, Lin et al. [21] found no connection between the radiotherapy dose prescribed and the risk of fracture after treatment for a soft tissue sarcoma (cohort of 205 patients), whereas Holt et al. [22] found a higher rate of fracture with prescribed doses of 60 or 66 Gy (20 patients out of 192, or 10.4%) than with a prescribed dose of 50 Gy (3 patients out of 172, or 1.7%).

After matching, Dickie et al. [23] reported a maximum dose of 64 Gy (1 SD = 7), an average dose of 45 Gy (1 SD = 8) and a V40 of 76% (1 SD = 17) in 21 patients suffering fractures after treatment by radiotherapy and surgery for a soft tissue sarcoma of the lower limbs, compared with 59 Gy (1 SD = 8), 37 Gy (1 SD = 11) and 64% (1 SD = 22), respectively, in 53 patients without fracture (*p* = 0.01, *p* = 0.02, *p* = 0.01, respectively). The authors thus recommended maintaining doses below these last three values in order to limit the risk of fracture. Lastly, the average dose on the fracture site was 59 Gy (1 SD = 7).

These data are summarized in the systematic review of Soares et al. [24].

In our cohort, the only patient with a proximal humerus fracture was aged 60 at the time of fracture, was receiving hormone therapy (letrozole then exemestane), and the fracture had occurred following a fall from a height 39 months after the end of radiotherapy. The treatment had involved a mastectomy, followed by irradiation of lymph node areas L2 + L3 + L4 + IMLN. The humeral head had received a maximum dose of 13.4 Gy and an average dose of 8.2 Gy. The role of radiotherapy in the occurrence of the fracture is therefore debatable.

This study does, however, present some limitations. First of all, the follow-up time, although lengthy, may not have been sufficient to reveal all the effects of radiotherapy. We note, however, that in the Holt study [22], the median timeline for occurrence of fractures was 41 months, with a median follow-up period of 50 months. In the Dickie study [23], it was 38 months, with an average follow-up duration of 107 months.

Moreover, we studied only the dose received by the humeral head. It is indeed possible that the clinical events studied are also linked to disorders of the soft tissue (muscle, ligaments, conjunctive tissue), the shoulder joint, the shoulder blade and the proximal part of the humerus. The humeral head alone would not be the most relevant area to predict the risk of occurrence of these clinical events.

Bazan et al. [25] studied the dose received by the shoulder, including the muscles (including all or part of the trapezium muscle, the levator scapulae muscle, the deep cervical flexors, the posterior scalene, the biceps bracchi, the deltoid, the subscapularis, the infraspinatus muscle, the large dorsal muscle and the large pectoral muscle), the soft tissues, bones, and vascularization. They did not, however, find any direct link between the dose received and the q-DASH score (quick disabilities of the arm, shoulder and hand) assessed at least 6 months from the end of treatment.

Johansen et al. [26] found a correlation between the V15Gy in the shoulder (including humerus coracoid and acromion processes) and the occurrence of stiffness, pain and limited joint movement. The maximum dose and the average dose were not associated with any clinical events.

Lipps et al. [27] studied the dose received by nine shoulder muscles in different treatment plans and showed that the V48 was the highest for the large and small pectoral muscles.

Third, we did not explore the role of the aromatase inhibitor, which can induce bone pain and functional impairment of multiple joints [28]. However, this side effect occurs in the majority of cases within 2 years after initiation of the treatment (frequency peak is 6 months) and usually disappears after 6–18 months [29]. As the bone pain reported here occurred later than that and specifically concerned the ipsilateral shoulder, it seemed unlikely to us that it might be a consequence of hormonotherapy.

Lastly, we did not include patients treated by 3D-CRT, in order to compare the dosimetric data.

Welgemoed et al. [30] compared the doses received by the humeral heads during classic conformational 3D radiotherapy and treatment via two dual-beam IMRT techniques with forward planning. They found a lower dose (V5 Gy, V10 Gy and V15 Gy) with the IMRT techniques. However, the rotational and inverse planning IMRT techniques, for which there are greater fears in terms of increased overall dose, were not studied. Similarly, Bazan et al. [25] found a reduction in V20–V50 Gy with treatment via an IMRT technique when compared with a conventional 3D technique, but the IMRT technique was not described, and the volume studied was not the same as in our study.

Lastly, Dogan et al. [20] found a maximum dose of 36.1 Gy, 54.3 Gy, 46.4 Gy, 45.8 Gy and 39.9 Gy, respectively, with treatment plans in 3D-CRT and IMRT with inverse planning with two, four, six and nine beams generated for 10 patients (statistically significant difference between 3D-CRT and IMRT with two, four and sixbeams). It was also shown [31] that good planning target volume (PTV) coverage could be obtained with six-beam IMRT and VMAT (V90% of 98.2% and 99.5%, respectively) despite the protection of the humeral head, while this PTV coverage was poor in 3D-CRT (V90% decreasing from 97.9% to 89.4%) when the V40Gy of the humeral head volume +10 mm was limited to <1cc.

## 5. Conclusions

This work has shown that there was no relationship between the dose received in the humeral heads and the pain, fractures and impaired mobility arising during follow-up after treatment by IMRT using tomotherapy for breast cancer patients. The humeral heads do not appear to receive a dose that is actually risky for the bony tissue. Humeral head delineation and protection of this at-risk organ may nonetheless be left to the clinician’s discretion, particularly in the event of irradiation of lymph node areas L1, L2 and L3, since the data remain piecemeal.

## Figures and Tables

**Table 1:** Factors significantly associated with the maximal dose received by the ipsilateral humeral head and description of maximal and mean doses in different subgroups. Gy: gray; CI: confidence interval; *n*: number of breasts.

Dosimetric Study	Maximal Dose (Gy): Mean (95%CI)	Mean Dose (Gy): Mean (95%CI)	*p*-Value for Difference in Maximal Dose
All breasts (*n* = 73)	24.41 (21.20–27.61)	9.18 (7.81–10.54)	
Lymph node level treated			
L4 + IMN (*n* = 12)	10.99 (7.49–14.49)	5.08 (3.72–6.44)	reference
L2 + L3 + L4 + IMN (*n* = 48)	24.72 (21.09–28.35)	9.40 (7.74–11.06)	<0.001
L1 + L2 + L3 + L4 + IMN (*n* = 13)	35.64 (27.62–43.66)	12.15 (7.99–16.30)	<0.0001
Side treated			
Right (*n* = 33)	28.24 (23.47–33.01)	10.44 (8.20–12.68)	reference
Left (*n* = 40)	21.25 (16.98–25.51)	8.14 (6.44–9.84)	0.03
Clinical event			
No (*n* = 63)	24.65 (21.20–28.10)	9.32 (7.87–10.77)	reference
Yes (*n* = 10)	22.90 (12.39–33.41)	8.29 (3.48–13.10)	0.74
Breast surgery			
Conservative (*n* = 62)	24.20 (20.65–27.75)	9.24 (7.73–10.76)	reference
Mastectomy (*n* = 11)	25.61 (16.91–34.31)	8.82 (5.14–12.50)	0.67
Age			0.62
BMI			0.19
Height			0.25

**Table 2:** Number of clinical events by subtypes (proximal humerus fracture, shoulder pain and functional limitation) and median time to the onset of symptoms.

Clinical Events	Number of Events	Median Time to Onset of Symptoms in Month (Min–Max)
All	10	48 (6–99)
Proximal humerus fracture	1	39
Shoulder pain	6	54 (21–99)
Functional limitation	3	45 (6–72)

Data in parentheses are ranges.

**Table 3:** Factors significantly associated with the occurrence of aclinical event. Gy: gray; *n*: number of breasts; IMN: internal mammary nodes; ALND: axillary lymph node dissection.

Clinical Study	Number of Clinical Events	Frequency of Clinical Event *	*p*-Value
Total (*n* = 165)	10	6.06% (3.20–10.92%)	
Lymph node level treated			
L4 + IMN (*n* = 23)	0	0% (0–14.8%)	reference
L2 + L3 + L4 + IMN (*n* = 111)	7	6.31% (2.87–12.66%)	0.60
L1 + L2 + L3 + L4 + IMN (*n* = 31)	3	9.68 % (2.56–25.69%)	0.27
Side treated			
Right (*n* =86)	4	4.65% (1.28–11.48%)	reference
Left (*n* = 79)	6	7.59% (2.83–15.8%)	0.53
Breast surgery			
Conservative (*n* = 134)	5	3.73% (1.22–8.49%)	reference
Mastectomy (*n* = 31)	5	16.12% (5.4–33.72%)	0.03
ALND			
Yes (*n* = 129)	10	7.75% (3.78–13.79%)	reference
No (*n* = 36)	0	0% (0–9.73%)	0.22
Diabetes			
Yes (*n* = 9)	1	11.11% (2.81–48.25%)	reference
No (*n* = 153)	9	5.88% (2.72–10.87%)	0.46
Age			0.21
BMI			0.09

* Data in parentheses are 95% confidence intervals.

## Data Availability

The data presented in this study are available on request from the corresponding author.
